# Tophaceous pseudogout of the temporomandibular joint extending into the cranium: a case report with literature review

**DOI:** 10.1093/jscr/rjac055

**Published:** 2022-03-07

**Authors:** Kei Takeda, Ikuya Miyamoto, Ryosuke Abe, Tadashi Kawai, Yu Ohashi, Hiroyuki Yamada

**Affiliations:** 1 Division of Oral and Maxillofacial Surgery, Department of Reconstructive Oral and Maxillofacial Surgery, School of Dentistry, Iwate Medical University; 2 Department of Dentistry and Oral Surgery, Aomori Prefectural Central Hospital; 3 Department of Dentistry and Oral Surgery, Iwate Prefectural Central Hospital

**Keywords:** pseudogout, cranium, temporomandibular joint

## Abstract

Pseudogout is a disease characterized by calcium pyrophosphate crystal deposition. Involvement of the temporomandibular joint (TMJ) is rare. We herein report a case of tophaceous pseudogout of the TMJ with cranial extension. An 83-year-old woman was referred to our institution for treatment of right TMJ pain. The patient’s medical and family histories were unremarkable. Magnetic resonance imaging showed a mass of about 35 mm in diameter compressing the bottom of the right temporal lobe of the brain. Based on a clinical diagnosis of a right TMJ tumour, biopsy was performed under general anaesthesia. The histopathological diagnosis was pseudogout. Considering the risk of surgically induced brain damage, the patient’s advanced age and her relatively good quality of life, the treatment plan simply involved the observation of the lesion. Fourteen months after biopsy, the patient’s activities of daily living remained unchanged and she had no TMJ pain.

## INTRODUCTION

Pseudogout is a disease characterized by calcium pyrophosphate crystal deposition, and it usually occurs in individuals older than 50 years. The most frequently involved joint is the knee, followed by the wrists, elbows, shoulders and ankles [1]. Involvement of the temporomandibular joint (TMJ) is rare; furthermore, only nine cases of pseudogout of the TMJ extending into the skull base have been reported in the English-language

**Figure 1:**
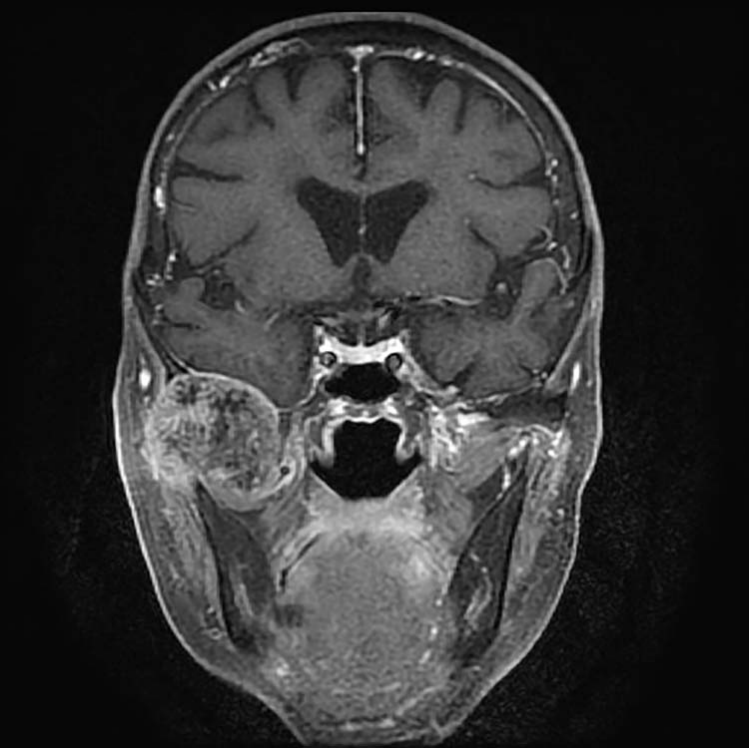
Gadolinium-enhanced *T*_1_-weighted magnetic resonance imaging showing the TMJ mass compressing the bottom of the right temporal lobe of the brain.

**Figure 2:**
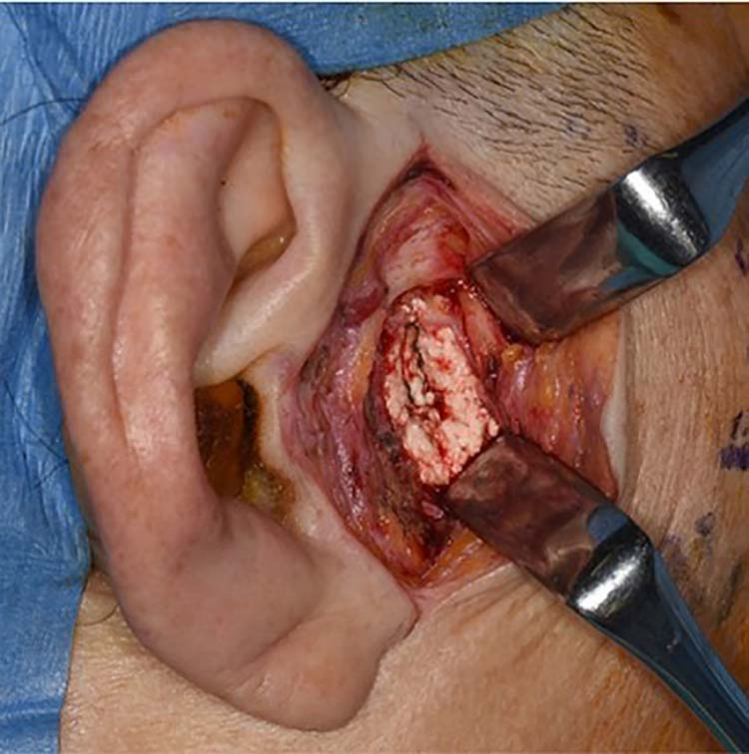
Intra-operative photograph showing a mass of white substance in the temporomandibular region.

**Figure 3:**
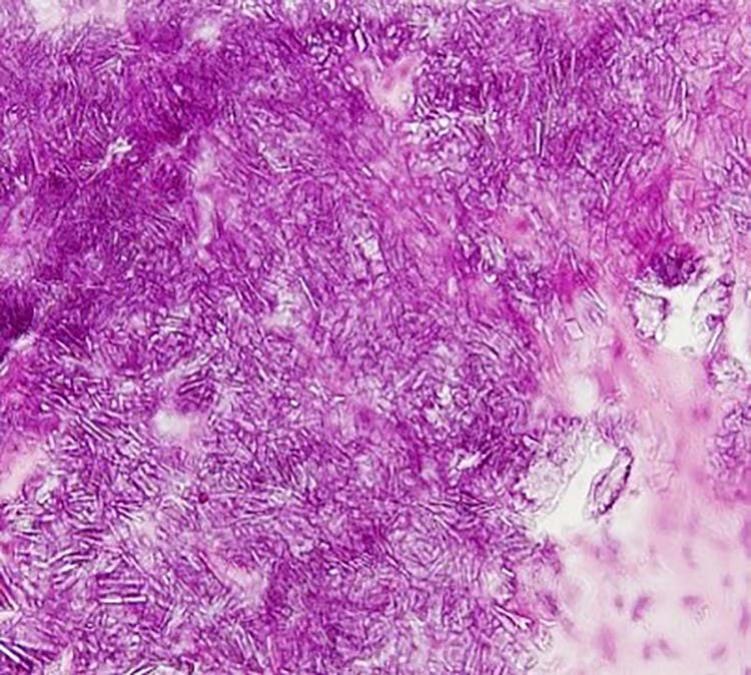
Photomicrograph showing lobular basophilic materials with variously sized rectangular or parallelogram-shaped crystals (haematoxylin–eosin stain, ×400).

**Figure 4:**
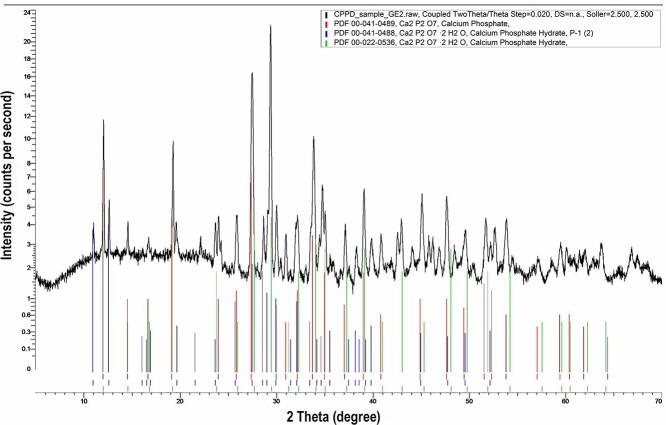
The result of X-ray diffraction analysis of the biopsy specimen showing the pattern of calcium pyrophosphate crystals.

literature [2–9]. Because of the rarity of this condition, the treatment outcome of such cases was not elucidated.

We herein report a case of large pseudogout of the TMJ with cranial extension and present a review of the literature.

## CASE REPORT

An 83-year-old woman was referred to our institution for treatment of right TMJ pain. She had first noticed the pain ~3 years earlier. The patient’s medical and family histories were unremarkable. Intra-oral examination revealed no abnormal findings associated with the right TMJ pain. Occlusal deviation was not observed. The maximum mouth opening was 28 mm, and transient pain occurred during mouth opening. A hard protrusion was observed in the right TMJ region. There was no evidence of cranial nerve paralysis or cervical lymphadenopathy. On *T*_1_- and *T*_2_-weighted magnetic resonance coronal images, the mass showed low signal intensity and compressed the bottom of the right temporal lobe of the brain. The mass about 35 mm in diameter was inhomogeneously enhanced by gadolinium (Fig. 1). The patient’s calcium, phosphate and uric acid concentrations were within the reference range.

Based on a clinical diagnosis of a right TMJ tumour, biopsy was performed under general anaesthesia (Fig. 2). Histopathological examination revealed lobular basophilic materials surrounded by fibrous tissue. Variously sized rectangular or parallelogram-shaped crystals were irregularly present within the basophilic materials (Fig. 3). These crystals were identified under polarized light.

The result of X-ray diffraction analysis of the biopsy specimen was consistent with the pattern of calcium pyrophosphate crystals (Fig. 4).

The final diagnosis of tophaceous pseudogout of the right TMJ was made based on these findings. A neurosurgical consultation in our hospital was performed. Considering the risk of surgically induced brain damage, the patient’s advanced age and her relatively good quality of life, the treatment plan simply involved the observation of the lesion with pain control. Fourteen months after biopsy, the patient was pain-free and her activities of daily living had remained unchanged.

**Table 1:** Reported cases of pseudogout of TMJ extending into the skull base

No	Author	Year	Age/sex	Symptoms	Size of the lesion (cm)	Image findings relating to skull base	Treatment	Post-operative complications	Follow-up periods
1	Grant [2]	1999	65/F	Facial fullness, discomfort, pain, facial swelling	4.8 × 5.6 × 6.5	Extending into the middle cranial fossa	Surgery	Not documented	6 w
2	Nicholas [3]	2007	35/M	External auditory canal tenderness	NA	Eroding into the middle cranial fossa	Surgery (partial superficial parotidectomy + infratemporal fossa dissection)	Not documented	NA
3	Kudoh [4]	2017	38/M	Mild pain in the chin and tip of the tongue, preauricular swelling	NA	Erosive bone resorption at the base of the skull	Observation after biopsy	No complication No change in size	36 m
4	Hotokezaka [5]	2020	59/F	Cheek swelling, pain, trismus	NA	Destroying the glenoid fossa	Surgery	No complication No mass recurrence	168 m
5	Abou-Foul [6]	2020	56/F	TMJ discomfort, swelling, trismus	2 × 3	Skull base erosion	Surgery (resection and total TMJ reconstruction)	No complication No mass recurrence	24 m
6	Houghton [7]	2020	55/F	Painless preauricular mass	2	Erosion into the middle cranial fossa	Surgery	No complication No mass recurrence	12 m
7	Tnag [8]	2021	46/F	Temporal swelling and pain, chewing discomfort	2 × 2	Destroying the glenoid fossa	Surgery (resection and arthroplasty)	No complication No mass recurrence	1 w
8	Tnag [8]	2021	52/M	Mass in the TMJ area pain and tinnitus	4 × 4	The mass infiltrated the middle cranial fossa	Surgery (resection and TMJ reconstruction)	No complication No mass recurrence	12 m
9	Morita [9]	2021	83/F	Cheek swelling	5 × 6	Erosion of mid-cranial fossa	Surgery	Conductive hearing loss	4 m
10	Present case		83/F	TMJ pain, trismus	3.7 × 3.3	The mass compressed the middle cranial fossa	Observation after biopsy	No complication slight increase in size	14 m

F: female, M: male, NA: not applicable, w: week, m: month.

## DISCUSSION

To the best of our knowledge, 10 cases (including the present case) of pseudogout of TMJ extending into the skull base have been reported in the English-language literature (Table 1). The most frequent clinical symptoms in these cases were swelling and pain in five patients and trismus in three patients. No patients had symptoms related to a central nervous system disorder. Pseudogout was pre-operatively diagnosed in 8 of the 10 patients. The remaining two patients were diagnosed with a neoplastic lesion or synovial osteochondromatosis [5] and synovial chondromatosis [7], respectively. General treatment of pseudogout is supportive to minimize symptoms [1]; however, tophaceous pseudogout that destroys surrounding structures sometimes requires surgery. Surgery was performed in six (75%) of the eight reported cases correctly diagnosed as pseudogout pre-operatively. Cerebrospinal fluid leakage occurred as an intra-operative complication in one patient [8]. Exposure of the dura mater was overlaid using a flap of temporal muscle [2, 5, 9], temporalis fascia [7], harvested fat [2] and bone wax [8]. No patients developed brain damage as a post-operative complication. However, conductive hearing loss was reported in one 83-year-old patient [9]. To avoid possible surgically induced complications, observation was selected in two patients, including ours. These patients experienced no deterioration of clinical symptoms within the follow-up period [4]. Because our patient’s oral dysfunction in daily life was mild, observation with pain control was selected.

## References

[1] Harvai A . Crystal-induced arthritis. In: Kumar V, Abbas AK, Aster JC (eds). Pathologic basis of disease, 10th edn. Philadelphia: Elsevier, 2021, 1204–7.

[2] Grant GA , Wener MH, Yaziji H, Futran N, Bronner MP, Mandel N, et al. Destructive tophaceous calcium hydroxyapatite tumor of the infratemporal fossa. Case report and review of the literature. J Neurosurg 1999;90:148–52. 10.3171/jns.1999.90.1.0148 10413170

[3] Nicholas BD , Smith JL 2nd, Kellman RM. Calcium pyrophosphate deposition of the temporomandibular joint with massive bony erosion. J Oral Maxillofac Surg 2007;65:2086–9. 17884544 10.1016/j.joms.2006.02.030

[4] Kudoh K , Kudoh T, Tsuru K, Miyamoto Y. A case of tophaceous pseudogout of the temporomandibular joint extending to the base of the skull. Int J Oral Maxillofac Surg 2017;46:355–9. 27641810 10.1016/j.ijom.2016.08.018

[5] Hotokezaka Y , Hotokezaka H, Katayama I, Fujita S, Sasaki M, Eida S, et al. A case of tophaceous pseudogout of the temporomandibular joint extending into the cranium. Oral Radiol 2020;36:203–8. 31559516 10.1007/s11282-019-00410-4

[6] Abou-Foul AK , Saeed NR. Treatment of calcium pyrophosphate deposition in the temporomandibular joint with resection and simultaneous reconstruction using a custom joint prosthesis. Oral Maxillofac Surg 2020;24:235–8. 31845090 10.1007/s10006-019-00825-7

[7] Houghton D , Munir N, Triantafyllou A, Begley A. Tophaceous pseudogout of the temporomandibular joint with erosion into the middle cranial fossa. Int J Oral Maxillofac Surg 2020;49:1286–9. 32278623 10.1016/j.ijom.2020.03.011

[8] Tang T , Han FG. Calcium pyrophosphate deposition disease of the temporomandibular joint invading the middle cranial fossa: two case reports. World J Clin Cases 2021;9:2662–70. 33889634 10.12998/wjcc.v9.i11.2662 PMC8040178

[9] Morita Y , Yamamoto N, Uchiyama T. Nodular pseudogout of the skull base arising from the temporomandibular joint. J Craniofac Surg 2021;32:e475–e7. 33741871 10.1097/SCS.0000000000007437

